# Can psychobiotics intake modulate psychological profile and body composition of women affected by normal weight obese syndrome and obesity? A double blind randomized clinical trial

**DOI:** 10.1186/s12967-017-1236-2

**Published:** 2017-06-10

**Authors:** Antonino De Lorenzo, Micaela Costacurta, Giuseppe Merra, Paola Gualtieri, Giorgia Cioccoloni, Massimiliano Marchetti, Dimitrios Varvaras, Raffaella Docimo, Laura Di Renzo

**Affiliations:** 1 Section of Clinical Nutrition and Nutrigenomic, Department of Biomedicine and Prevention, University of Rome Tor Vergata, 00133 Rome, Italy; 2 Department of Experimental Medicine and Surgery, University of Rome “Tor Vergata”, Rome, Italy; 3 Emergency Department, “A. Gemelli” General Hospital Foundation, Catholic University of Sacred Heart, 00168 Rome, Italy; 4 PhD School of Applied Medical-Surgical Sciences, University of Rome “Tor Vergata”, 00133 Rome, Italy; 5 USL 1 UmbriaCastiglione del Lago, 06061 Perugia, Italy; 6 Department of Surgical Sciences, University Hospital “Umberto I”, “Sapienza” University of Rome, 00161 Rome, Italy; 7 Department of Experimental Medicine and Surgery, University of Rome Tor Vergata, 00133 Rome, Italy; 8 Section of Clinical Nutrition and Nutrigenomic, Department of Biomedicine and Prevention, University of Rome Tor Vergata, Via Montpellier 1, 00136 Rome, Italy

**Keywords:** NWO syndrome, Probiotic, Psychobiotic, Psychological profile, Body composition

## Abstract

**Background:**

Evidence of probiotics effects on gut function, brain activity and emotional behaviour were provided. Probiotics can have dramatic effects on behaviour through the microbiome–gut–brain axis, through vagus nerve. We investigated whether chronic probiotic intake could modulate psychological state, eating behaviour and body composition of normal weight obese (NWO) and preobese–obese (PreOB/OB) compared to normal weight lean women (NWL).

**Methods:**

60 women were enrolled. At baseline and after a 3-week probiotic oral suspension (POS) intake, all subjects underwent evaluation of body composition by anthropometry and dual X-ray absorptiometry, and psychological profile assessment by self-report questionnaires (i.e. EDI-2, SCL90R and BUT). Statistical analysis was carried out using paired t test or a non-parametric Wilcoxon test to evaluate differences between baseline and after POS intake, one-way ANOVA to compare all three groups and, where applicable, Chi square or t test were used to assess symptoms.

**Results:**

Of the 48 women that concluded the study, 24% were NWO, 26% were NWL and 50% were PreOB/OB. Significant differences in body composition were highlighted among groups both at baseline and after a POS (p < 0.05). After POS intake, a significant reduction of BMI, resistance, FM (kg and %) (p < 0.05), and a significant increase of FFM (kg and  %) (p < 0.05) were observed in all subjects in NOW and PreOB/OB. After POS intake, reduction of bacterial overgrowth syndrome (p < 0.05) and lower psychopathological scores (p < 0.05) were observed in NWO and PreOB/OB women. At baseline and after POS intake, all subjects tested were negative to SCL90R_GSI scale, but after treatment subjects positive to BUT_GSI scale were significantly reduced (8.33%) (p < 0.05) compared to the baseline (33.30%). In NWO and PreOB/OB groups significant differences (p < 0.05) in response to the subscales of the EDI-2 were observed. Significant improvement of the orocecal transit time was observed (p < 0.05) after POS intake. Furthermore, significant differences were observed for meteorism (p < 0.05) and defecation frequency (p < 0.05).

**Conclusions:**

A 3-week intake of selected psychobiotics modulated body composition, bacterial contamination, psychopathological scores of NWO and PreOB/OB women. Further research is needed on a larger population and for a longer period of treatment before definitive conclusions can be made.

*Trial registration* ClinicalTrials.gov Id: NCT01890070

## Background

The human gut hosts a dynamic and complex microbial ecosystem. It is represented by approximately 1 kg of bacteria in the average adult, about the weight of the human brain. It is composed by microorganisms belonging to 14 families, 45 genera and 400–500 distinct species, variously distributed along the entire intestinal tract. Two bacterial phylotypes *Firmicutes* and *Bacteroidetes* are the most abundant bacteria species in the gut [1].

The microbiota–gut brain axis, recently termed “psychobiota” [2], plays an important role in host physiology, as in the regulation of neuroinflammation, neuroendocrine stress response, neurodevelopment and modulation of mood and behavior [3–5], which implicates it in psychiatric disorders, such as stress, anxiety and depression.

Nowadays, several studies identify the relationship between gut microbiota and the onset of various diseases, demonstrating the ability of probiotics in controlling inflammatory processes, attenuation of metabolic dysfunctions, normalization of stress-induced abnormal behaviors, regulation of the hypothalamus–pituitary–adrenal axis (HPA axis) and neuropsychiatric disorders [6–8].

The evidence of the inflammatory state alteration, highlighted in schizophrenia, major depressive disorder and bipolar disorder, strongly recalls the microbiota alteration, suggesting an important role of the alteration of the gastrointestinal (GI) system also in neuropsychiatric disorders [8].

Probiotics can have dramatic effects on behaviour, through their action on the vagus nerve and on the microbiome–gut–brain axis, which constitutes a bidirectional communication network. Probiotics produce a variety of neurochemicals, analogues of mammalian hormones, involved in mood and behaviour. Therefore, the visceral messages from the gut can affect brain function and, vice versa, signals from the brain may affect the sensory system and the gut secretion mode.

The bacteria most commonly exploited as probiotics with psychotropic effects in animal models and human clinical trials have been classified as “psychobiotics” [2], and they belong to the *Bifidobacterium* and *Lactobacillus* families.

In the last few years, in vivo experiments demonstrated that administration of *Bifidobacterium longum* 1714 and *Bifidobacterium breve* 1205, *Lactobacillus helveticus and Lactobacillus plantarum strain PS128* reduce anxiety and depression like behaviors, HPA axis hyperactivity and abnormal neurochemical changes [9].

Gut microbiota seems to have an impact on the serotonergic system. In fact, treatment with *Lactobacillus helveticus* in hyperammonemia-treated rats led to an anxiolytic effect and improved cognitive function possibly through a reduction in hippocampal 5-Hydroxy Tryptamine (5HT) levels [10].

Moreover, a combined treatment with *Lactobacillus helveticus* and *rhamnosus* prevented intestinal permeability alteration caused by stress [11].

Anxiety-like behavior associated with chronic colitis in mice was attenuated by *B. longum* administration, associated with decreased hippocampal brain-derived neurotrophic factor (BDNF) mRNA [12, 13].

Although less evidence exists for probiotic actions on human population, it has been demonstrated that patients who received a probiotic formulation containing *Lactobacillus acidophilus*, *Lactobacillus casei* and *Bifidobacterium bifidum*, significantly decreased Beck Depression Inventory (BDI) scores [14]. Moreover, a probiotic mixture of *Lactobacillus helveticus* and *Bifidobacterium longum* and a probiotic-containing milk drink of *Lactobacillus casei* showed positive effects on psychological distress [15, 16].

Furthermore, a growing body of scientific evidence supports the notion that the crosstalk between the gut microbiota, diet and immune system activates mediators and signalling pathways, which influence the whole body metabolism and disease [17–19]. The metabolic activities of the gut microbiota play a decisive part in obesity because of its role in the improvement of calorie extraction from food, the accumulation of substrates in adipose tissue, like fatty acids, and the utilization of energy and nutrients for its growth [20].

Obesity is characterized by a peculiar microbiota and the microbiota itself, together with the host genotype and lifestyle, could contribute to the development of this metabolic dysfunction. Also, a bidirectional association between obesity and self-reported or clinical measures of depression were observed, and body dissatisfaction was robustly associated as a risk factor for obesity and eating disorders [9].

Different obese phenotypes have been described based on body fat composition and distribution, rather than the simple increase of body weight, body mass index (BMI) and genetics [21]: (1) metabolically obese normal weight [22]; (2) metabolically healthy obese [23]; and (3) metabolically unhealthy obese or “at risk” obese [24]; (4) normal weight obese (NWO) [25].

Subjects affected by NWO syndrome usually have a BMI value within the normal range (<25 kg/m^2^) and, at the same time, high total body fat percentage (TBFat >30%) and total body lean mass (TBLean) deficiency based on a genetic predisposition. They also have high oxidative stress level, early inflammatory status and some metabolic abnormalities [26–28]. Furthermore, in our previous study on female subjects affected by NWO syndrome it was highlighted, not only an increased risk of cardiovascular and metabolic disease, but also a will to control body weight, revealing a suppressed vocation for obesity. In fact, NWO subjects obtained an intermediate score on the eating disorder inventory-2 (EDI-2), particularly in terms of body dissatisfaction and drive for thinness, between normal weight lean women and pre-obese or obese women [28].

Up today very few studies demonstrated the beneficial effects on the health status of obese subjects with psychiatric illnesses and eating disorders of these “mind-altering” probiotics. *Lactobacillus* appears to reduce body fat mass, anxiety and dysphoria, and improves insulin sensitivity and glucose tolerance [29, 30].

Nowadays, literature doesn’t report any paper focused on the effectiveness check of treatment with psychobiotics in NWO syndrome.

Given the link among gut microbiota, body composition, the risk of eating disorder, anxiety- and depression-like behaviours, the purpose of the current study was to test the efficacy of a 3-week administration of a psychobiotic oral suspension (POS), containing *Streptococcus thermophilus*, *Bifidobacterium animalis* subsp. *Lactis*, *Streptococcus thermophiles*, *Bifidobacterium bifidum*, *Lactobacillus delbrueckii* spp. *Bulgaricus, Lactococcus lactis* subsp. *Lactis, Lactobacillus acidophilus, Lactobacillus plantarum, Lactobacillus reuteri*, selected according to literature. The endpoints of this study were to evaluate the body composition parameters, the psychological profile, and eating behaviour changes of NWO and preobese–obese (PreOB/OB) compared to normal weight lean women (NWL), after POS.

## Methods

### Clinical study design and subjects

**Fig. 1:**
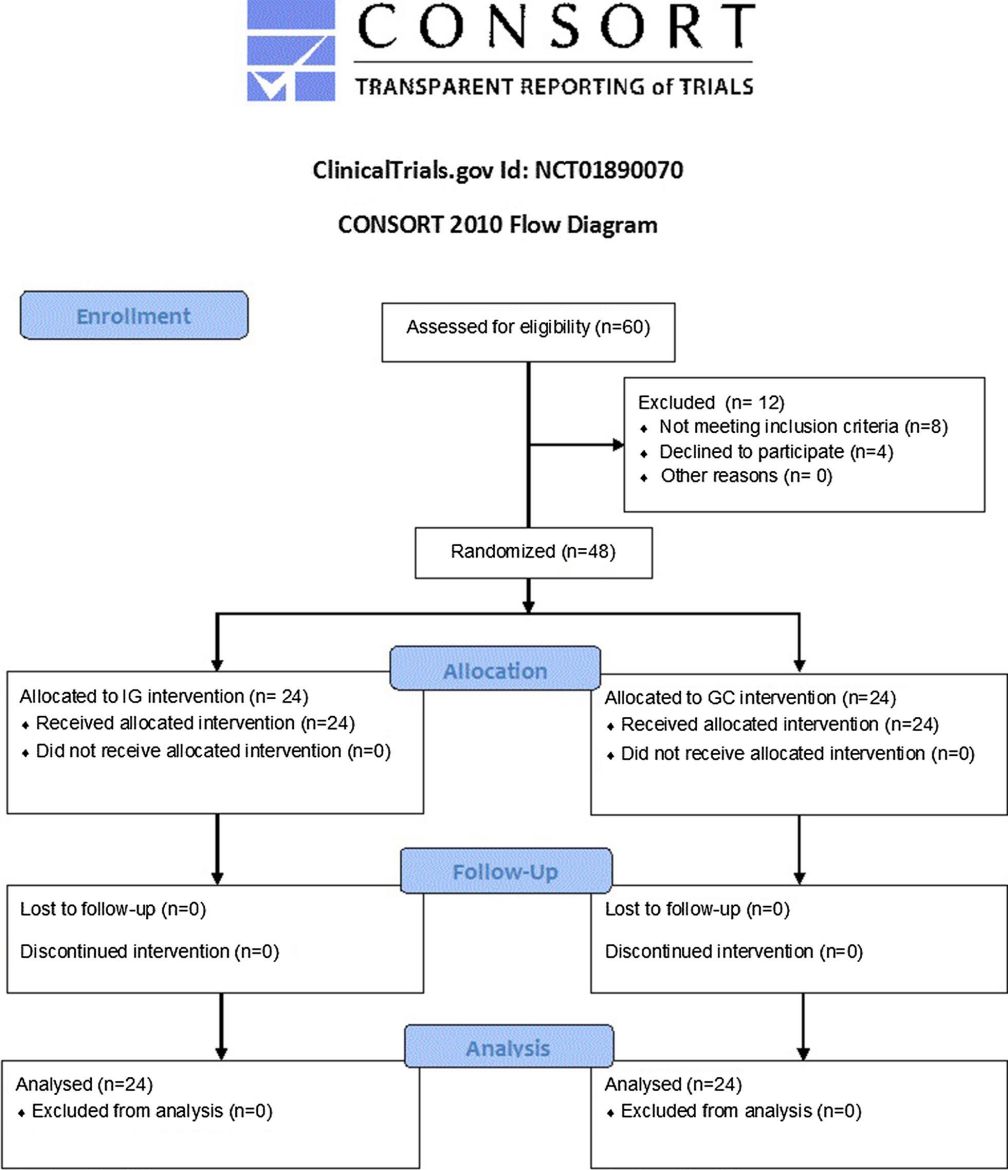
Study Flow Diagram according to Consort, 2010

The clinical study was conducted using a randomized, double blinded controlled design, between October 2015 and July 2016, according to the CONSORT flowchart (Fig. 1). Subjects were consecutively recruited within a program of routine medical check-up at the Section of Clinical Nutrition and Nutrigenomic, Department of Biomedicine and Prevention of the University of Rome “Tor Vergata”, at “Nuova Annunziatella” Clinic, and General Hospital Foundation, Catholic University of Sacred Heart, Rome, Italy.

Sixty women were enrolled. Subjects were screened for eligibility at first medical visit and underwent body composition analysis, psychological profile and eating behaviour assessment by symptom checklist 90 (SCL90R), [31] for the evaluation of general psychopathology, body uneasiness test (BUT), for the evaluation of body image perception (IC) [32], and EDI-2, for eating behavior [33], in order to test the efficacy of a 3-week administration of POS on NWO and preobese–obese (PreOB/OB) compared to normal weight lean women (NWL).

As the expected weight loss was below 5, according the Ethical Committee of the University of Rome “Tor Vergata”, data from Dual X-ray Absorptiometry (DXA) was used to classified the subjects. We classified the subjects according to BMI, and TBFat % by DXA into: (a) NWL women, with a BMI <25 kg/m^2^ and TBFat % <30; (b) NWO women, with a BMI <25 kg/m^2^, and TBFat % ≥30; (c) PreOB/OB women, with a BMI ≥25 kg/m^2^ and TBFat % ≥30.

Body composition was assessed by Bioelectrical Impedance Analysis (BIA) at baseline and after treatment.

Forty-eight subjects were eligible for the study and they were randomly divided into two groups (1:1 ratio). The randomization was determined by an external contract research organization and coordinated with the Section of Clinical Nutrition and Nutrigenomic, at the University of Rome “Tor Vergata”, independently of the investigators.

One intervention group (IG) and one control group (CG) were utilized and took daily n.1 bag of POS, or n.1 bag of placebo. The study consists of a 3-week treatment, with POS or placebo, separated by a 3-week wash out period, used to avoid additive effects on treatments to follow. After 3 weeks of washout, the IG and CG were reversed. The IG and CG arms were double-blinded. Study period resulted in a total duration of 9 weeks. At the beginning and at the end of each arm (±3 days), the subjects had to repeat the visit.

Subjects were asked to maintain their usual lifestyle habits and to report any illness or abnormality arising during the study. At the end of each arm, a clinician assessed any adverse effects from the interventions by going through a checklist of symptoms, including bloating, fullness or indigestion, altered bowel habit, dizziness and other symptoms that were possibly associated with the interventions. Any adverse effect has been properly reported.

### Exclusion criteria

Exclusion criteria included age <20 or >65 year, pregnancy, breast-feeding, type 1 diabetes, presence of intestinal bacterial overgrowth, characterized by high levels of hydrogen and methane production in the small bowel, acute diseases, endocrine disorders, liver, heart or kidney dysfunctions, history of chronic degenerative or infectious diseases, medication, antibiotic therapy until 10 days before enrolment, smoke, drug or alcohol abuse, participation in another diet trial. No subjects with known alterations to intestinal transit following organic pathologies (abdominal surgery, diabetes mellitus, scleroderma, hypothyroidism, etc.) were included in the study. Subjects could not have taken antibiotics or probiotics in the month before the study and were willing to avoid use of probiotics for the duration of the study.

### Endpoints

The primary endpoint was the evaluation of body composition changes after POS by anthropometry, DXA, and bioimpedentiometry. The secondary endpoint was the evaluation of psychological profile by self-report questionnaires (i.e. EDI-2, SCL90R, and BUT). The third endpoint was the evaluation of orocaecal transit time and bacterial overgrowth by lactulose breath test (LBT).

### Psychobiotics oral suspension (POS) and placebo capsules composition

1 bag of 3 g psychobiotics oral suspension (POS) contained: *Streptococcus thermophilus SGSt01* (1.5 × 10^10^ colony-forming unit CFU), *Bifidobacterium animalis* subsp. *Lactis* SGB06 (1.5 × 10^10^ colony-forming unit CFU), *Streptococcus thermophiles* (1.5 × 10^10^ colony-forming unit CFU), *Bifidobacterium bifidum* SGB02 (1.5 × 10^10^ colony-forming unit CFU), *Lactobacillus delbrueckii* spp. *Bulgaricus DSM 20081* (1.5 × 10^10^ colony-forming unit CFU)*, Lactococcus lactis* subsp. *Lactis SGLc01* (1.5 × 10^10^ colony-forming unit CFU)*, Lactobacillus acidophilus SGL11* (1.5 × 10^10^ colony-forming unit CFU)*, Lactobacillus plantarum SGL07* (1.5 × 10^10^ colony-forming unit CFU)*, Lactobacillus reuteri* SGL01 (1.5 × 10^10^ colony-forming unit CFU), maltodextrin from corn, anti-caking agent (silica), casein, lactose and gluten <3 ppm LLOQ (lower limit of quantitation) (Biocult strong, HOMEOSYN, Rome, Italy).

The placebo was represented by 3 g of inert material (flour type 00), maltodextrin from corn, anti-caking agent (silica). The appearance of the placebo was indistinguishable in colour, shape, size, packaging, smell, and taste from that of the probiotic supplement.

Both products were provided by HOMEOSYN (Rome, Italy).

#### Anthropometric measurements

At T1, after a 12-h overnight fast, all subjects underwent anthropometric evaluation (body weight, height, waist and hip circumferences), according to standard method [34]. All the individuals were instructed to take off their clothes and shoes before undergoing the measurements.

BMI was calculated using the formula: BMI = body weight (kg)/height (m)^2^.

#### Bioelectrical impedance analysis (BIA)

Resistance, reactance, impedance, phase angle, total body water (TBW), intracellular water (ICW), extracellular water (ECW), free fat mass (FFM) and fat mass (FM) were measured using a BIA phase sensitive system at 50 kHz frequency (BIA 101S, Akern/RJL Systems-Florence, Italy).

Measurements were taken according to Di Renzo et al. [35].

#### Dual x-ray absorptiometry

To assess body composition analysis, that give the possibility to measure TBFat and TBLean, DXA (i-DXA, GE Medical Systems, Milwaukee, WI, USA) evaluation was performed at baseline, according to De Lorenzo et al. [28].

#### Psychodiagnostic instruments

Anonymous questionnaires, self-compiled, were given to all subjects for the collection of socio-demographic data. The symptom checklist 90 (SCL90R) [31] was administered for the evaluation of general psychopathology, the body uneasiness test (BUT) for the evaluation of the perception of body image (IC) [32] and the EDI-2, for eating behavior [33]. Eating behavior was assessed using the Italian version of the EDI-2, standardized in an Italian population [36]. The subscales of the EDI-2 include drive for thinness (DT), bulimia (B), body dissatisfaction (BD), ineffectiveness (I), interceptive awareness (IA), maturity fears (MF), asceticism (A), impulse regulation (IR), social insecurity (SI), perfectionism (P) and interpersonal distrust (ID).

##### Body uneasiness test (BUT)

It is a self-assessment scale, used for body image studies and related pathologies. Beyond the total score, BUT allows to calculate the global severity index (GSI) or total average score, which is obtained from the sum of clinical scores (BUT a), divided by their number (34). Items number with score ≥1 correspond to positive symptom total (PST). The sum of items scores ≥1 divided by PST, produces the positive symptom distress index (PSDI) [32].

Five factors were defined: WP—weight phobia, BIC—body image concerns, A—avoidance, CSM—compulsive self-monitoring, D—depersonalization. In our study, we considered as positive for altered perception of body image a GSI score ≥1.2.

##### Symptom check list—revised (SCL90R)

It is a general evaluation scale of the psychopathology, based on patient’s self-evaluation. This scale is composed by 90 items, which investigate the presence of symptoms in the week before the test check. These 90 items, which have five levels Likert answers, have 10 reference factors: (1) somatization (Som); (2) obsessive/compulsive (Obs); (3) interpersonal sensitivity (Interp Sens); (4) depression (Dep); (5) anxious (Anx); (6) anger/hostility (Anger Host); (7) phobia (Phob); (8) psychoticism (Psych); (9) paranoia (Paran); (10) sleep disorders. The score goes from 0 to 4, and a score above 1 is an index of pathology [31].

### *Breath testing* and gastrointestinal symptoms questionnaire

Subjects also completed a questionnaire evaluating gastrointestinal symptoms (meteorism, abdominal pain, defecation frequency/week).

The LBT was performed by administering 20 g lactulose dissolved in 100 cc water to the subjects. Breath samples were obtained by asking the patients to blow into suitable containers at time 0 (before ingesting the lactulose) and then every 15 min thereafter for the 4 h following lactulose administration. Gas chromatography was used to assess the presence and quantity of hydrogen in the breath (Quintron Milwaukee, Wisconsin USA). Orocaecal transit time was calculated for each patient by constructing the curves of hydrogen in the breath over time. This therefore showed the time necessary for the bolus to reach the caecum [37].

#### Statistical analysis

The statistical analysis was carried out using IBM SPSS Statistics for Windows, Version 21.0 (Armonk, NY: IBM Corp). Data are expressed as mean ± standard deviation (SD), and minimum and maximum. A paired t test or a non-parametric Wilcoxon test were performed to evaluate differences between baseline and after POS. A one-way ANOVA was carried out to compare the average of the responses obtained in all three groups. Where applicable, the Chi square or Student’s t test were used to assess symptoms.

A difference of p < 0.05 was considered significant.

## Results

Of the 60 women initially recruited, 8 did not meet inclusion criteria, 4 dropped out of the study voluntarily, leaving a total of 48 subjects for final analysis.

The characteristics of the study population in terms of age, weight, height, BMI, body composition and psychological profile (EDI-2, BUT, and SCL90R) are shown in Table 1. In particular, between the NWL and the NWO groups, significant differences (p < 0.05), in terms of weight (Δ% 14.31), BMI (Δ% 16.95), hip circumference (Δ% 9.4), TBW (%) (Δ% −10.05), TBFat (%) (Δ% 32.77), TBFat (g) (Δ% 43.43), FM (kg) (Δ% 58.59), FM (%) (Δ% 39.75) and FFM (%) (Δ% −10.13) were observed. Between the NWL and the PreOB/OB there were significant differences (p < 0.05) in terms of weight (Δ% 25.06), BMI (Δ% 35.22), waist (Δ% 18.93) and hip circumference (Δ% 14.50), PA (°) (Δ%17.46), TBW (%), (Δ% −17.49), ECW (%) (Δ% −9.57), ICW (L) (Δ% 11.61), ICW (%) (Δ% 7.65), TBFat (%) (Δ% 64.97), TBFat (g) (Δ% 125.08), FM (kg) (Δ% 64.32), FM (%) (Δ% 43.35) and FFM (%) (Δ% −11.04). Significant differences (p < 0.05) between the NWO and the PreOB/OB groups in terms of weight (Δ% 9.40), BMI (Δ% 15.62), Reactance (Ohm), (Δ% 19.73), PA (°) (Δ% 20.72), TBW (L) (Δ% −0.60), TBW (%) (Δ% −8.27), ECW (L) (Δ% −12.45), ECW (Δ% −11.70), and ICW (L) (Δ% 9.50), ICW (%) (Δ% 9.78), TBFat (%) (Δ% 24.26) and TBFat (g) (Δ% 56.93) were observed (Table 1).

**Table 1:** Comparison of body composition parameters of normal weight lean, normal weight obese and pre-obese/obese groups between baseline and after 3 weeks POS treatment

	NWL		NWO		PreobOB	
	Baseline	POS	Baseline	POS	Baseline	POS
Age (years)	30.18 ± 2.04 (28.00–33.00)		40.00 ± 12.56 (27.00–56.00)		33.57 ± 10.57 (24.00–50.00)	
Height (cm)	164.64 ± 4.03 (160.00–170.00)		162.90 ± 6.60 (154.00–168.00)		158.33 ± 1.86 (155.60–160.00)	
Weight (kg)	55.55 ± 4.65^¤,^° (50.50–62.50)	54.84 ± 5.63^Ϫ,ϣ^ (49.20–63.00)	63.50 ± 3.97^¥^ (59.00–68.30)	63.10 ± 3.27 (59.00–66.60)	69.47 ± 6.07 (63.80–78.00)	63.35 ± 0.16^b^ (63.20–63.50)
BMI (kg/m^2^)	20.47 ± 1.04^¤,^° (19.48–22.41)	20.49 ± 1.40^Ϫ,ϣ^ (18.98–22.59)	23.94 ± 0.93^¥^ (22.74–24.88)	23.80 ± 0.84^a,Ϭ^ (22.92–24.88)	27.68 ± 1.92 (25.56–30.47)	25.78 ± 0.48^b^ (25.32–26.23)
Waist (cm)	67.41 ± 4.22 (64.50–75.00)°	67.98 ± 4.60^Ϫ,ϣ^ (63.80–75.00)	74.25 ± 5.08 (69.50–79.00)	75.33 ± 4.70 (70.00–81.00)	80.17 ± 8.02 (73.00–91.00)	73.75 ± 2.35^b^ (71.50–76.00)
Hip (cm)	94.09 ± 4.57 ^¤,^° (87.00–97.00)	93.23 ± 4.35 ^Ϫ,ϣ^ (87.50–97.00)	103.25 ± 5.61^¥^ (98.00–108.50)	101.00 ± 3.72^Ϭ^ (98.00–106.00)	107.73 ± 0.54 (107.00–108.20)	106.75 ± 0.78^b^ (106.00–107.50)
Resistance (Ohm)	595.89 ± 85.36 (501.00–735.00)	598.33 ± 69.50 ^ϣ^ (497.00–690.00)	576.67 ± 55.46 (526.00–650.00)	567.00 ± 53.60^a^ (513.00–636.00)	565.13 ± 35.34 (526.00–611.00)	523.00 ± 24.02^b^ (500.00–546.00)
Reactance (Ohm)	66.00 ± 12.24 (56.00–87.00)	65.89 ± 8.46 (56.00–73.00)	61.33 ± 7.74^¥^ (53.00–71.00)	59.67 ± 8.75^a,Ϭ^ (51.00–71.00)	73.43 ± 9.54 (66.00–89.00)	69.50 ± 6.79 (63.00–76.00)
PA (°)	6.30 ± 0.35° (5.90–6.80)	6.29 ± 0.46^ϣ^ (5.70–6.80)	6.13 ± 0.89^¥^ (5.30–7.30)	6.03 ± 0.97^a,Ϭ^ (5.10–7.30)	7.40 ± 0.59 (6.70–8.30)	7.55 ± 0.37^b^ (7.20–7.90)
TBW (L)	31.80 ± 2.83 (29.10–36.60)	31.66 ± 3.00 (29.70–36.90)	33.13 ± 2.50^¥^ (31.10–36.50)	33.40 ± 2.56^a^ (31.10–36.80)	32.93 ± 0.36 (32.60–33.50)	33.35 ± 1.10 (32.30–34.40)
TBW (%)	57.92 ± 4.19^¤,^° (50.80–61.30)	57.82 ± 3.19 (52.40–60.40)	52.10 ± 1.37^¥^ (50.30–53.40)	52.90 ± 2.01^a^ (50.60–55.30)	47.79 ± 4.30 (42.00–52.60)	52.65 ± 1.83^b^ (50.90–54.40)
ECW (L)	14.11 ± 1.33 (12.40–16.10)	14.07 ± 1.39 (12.60–16.10)	15.10 ± 2.01^¥^ (12.50–17.10)	15.37 ± 2.18^a,Ϭ^ (12.50–17.40)	13.22 ± 0.74 (12.10–14.00)	13.20 ± 0.84^b^ (12.40–14.00)
ECW (%)	44.42 ± 1.38° (42.50–46.00)	43.93 ± 2.64^ϣ^ (40.80–47.20)	45.53 ± 3.93^¥^ (40.40–49.30)	45.87 ± 4.29^a,Ϭ^ (40.30–50.10)	40.17 ± 2.02 (37.10–42.60)	39.50 ± 1.36^b^ (38.20–40.80)
ICW (L)	17.66 ± 1.63° (16.70–20.50)	17.59 ± 1.86^ϣ^ (16.20–20.80)	18.00 ± 1.45^¥^ (16.10–19.40)	18.03 ± 1.47^a, Ϭ^ (16.10–19.40)	19.71 ± 0.63 (18.90–20.60)	20.15 ± 0.26^b^ (19.90–20.40)
ICW (%)	55.58 ± 1.38° (54.00–57.50)	55.62 ± 1.91^ϣ^ (52.92–57.58)	54.50 ± 3.91^¥^ (50.70–59.60)	54.12 ± 4.37^a,Ϭ^ (49.85–59.81)	59.83 ± 2.02 (57.40–62.90)	60.61 ± 1.37^b^ (59.30–61.92)
TBFat (%)	26.55 ± 2.83^¤,^° (24.10–29.00)	–	35.25 ± 1.44^¥^ (33.90–36.60)	–	43.80 ± 0.00 (43.80–43.80)	–
TBFat (kg)	15.15 ± 0.01^¤,^° (15.14–15.16)	–	21.73 ± 1.73^¥^ (20.11–23.34)	–	34.10 ± 0.00 (34.10–34.10)	–
TBLean (kg)	40.17 ± 5.98 (34.99–45.35)	–	37.61 ± 0.57 (37.08–38.14)	–	41.26 ± 0.00 (41.26–41.26)	–
FM (kg)	11.35 ± 2.86^¤,^° (8.10–15.10)	11.80 ± 3.12^c,Ϫ,ϣ^ (8.10–16.00)	18.00 ± 1.64 (15.90–19.70)	17.27 ± 1.80^a^ (15.80–19.70)	18.65 ± 0.99 (17.70–19.60)	16.95 ± 0.99^b^ (16.00–17.90)
FM (%)	20.30 ± 4.06^¤,^° (16.10–26.40)	21.05 ± 4.43^c,Ϫ,ϣ^ (16.50–27.80)	28.37 ± 2.09 (26.90–31.20)	27.57 ± 2.58^a^ (25.00–30.90)	29.10 ± 1.36 (27.80–30.40)	26.70 ± 1.46^b^ (25.30–28.10)
FFM (kg)	44.23 ± 3.57 (42.20–50.00)	43.75 ± 4.06 (41.10–50.30)	45.50 ± 3.25 (43.10–49.90)	45.83 ± 3.32^a^ (43.20–50.30)	45.50 ± 0.63 (44.90–46.10)	46.40 ± 0.84^b^ (45.60–47.20)
FFM (%)	79.70 ± 4.06^¤,^° (73.60–83.90)	78.95 ± 4.43^c,Ϫ,ϣ^ (72.20–83.50)	71.63 ± 2.09 (68.80–73.10)	72.43 ± 2.58^a^ (69.10–75.00)	70.90 ± 1.36 (69.60–72.20)	73.30 ± 1.46^b^ (71.90–74.70)

Results are expressed in mean value ± standard deviation, and minimum and maximum for each parameter. Values of p < 0.05 are considered significant

*BMI* body mass index, *ECW* extracellular water, *FM* fat mass, *FFM* fat free mass, *ICW* intracellular water, *NWL* normal weight lean, *NWO* normal weight obese, *PA* phase angle, *PreOB/OB* preobese–obese, *TB* total body, *TBW* total body water

^a^NWO T0 vs T1 p < 0.05; ^b^ PreOB/OB T0 vs T1 p < 0.05 ^c^ NWL T0 vs T1. For Anova-test at baseline p < 0.05; ^¤^ NWL vs NWO p < 0.05; ^¥^ NWO vs PreOB/OB p < 0.05; ° NWL vs PreOB/OB p < 0.05. Anova-test after POS p < 0.05; ^Ϫ^ NWL vs NWO p < 0.05; ^Ϭ^ NWO vs PreOB/OB p < 0.05; ^ϣ^ NWL vs PreOB/OB p < 0.05

After 3 weeks of POS treatment, a significant reduction of BMI, resistance, reactance, PA, ICW (%), FM (kg and %) (p < 0.05), and a significant increase of TBW (L and %), ECW (L and %), ICW (L), FFM (kg) and FFM (%) were observed in NOW population. Furthermore, in PreobOB group it was highlighted a significant reduction of weight, BMI, waist, hip, resistance, ECW (L and %), FM (kg and %), and a significant increase of PA, TBW (%), ICW (L and %), FFM (kg and %) (p < 0.05) (Table 1).

Significant differences (p < 0.05) were observed among the NWL, NWO and PreOB/OB groups in body composition parameters after POS treatment (Table 1).

At baseline, the total tested sample was negative to SCL90R_GSI scale and 33.30% of the population was positive at BUT_GSI scale (GSI ≥1.2; mean 0.96 ± standard deviation 0.66). After 3 weeks of POS treatment, all population remained negative to SCL90R_GSI scale, and the positive to BUT scale was significantly reduced (p < 0.05) at 8.33% (GSI ≥1.2; mean 0.59 ± standard deviation 0.52). At baseline, among the 33.3% of the positive to BUT_GSI and BUT_CSM scale, the 12.5% were NWL, the 50% were NWO, and 37.55% were PreOB/OB. After POS treatment only NWO among the 8.33% of the positive (100%) were identified.

The average scores of the various dimensions and the total score of SCL90R scale, BUT_GSI and EDI-2 are represented in Table 2. After 3 weeks of POS treatment, in the general population significant differences (p < 0.05) in terms of the responses to the subscales of the EDI-2 were observed: −37.98 Δ% of B (baseline, 1.74 ± 3.01; 3 weeks POS, 1.08 ± 2.06), the −15.95 Δ% of DT (baseline, 1.74 ± 3.01; 3 weeks POS, 1.08 ± 2.06), the −40.15 Δ% of I (baseline, 1.74 ± 3.01; 3 weeks POS, 1.08 ± 2.06). After POS treatment, in the NWO group significant differences (p < 0.05) in terms of the responses to the subscales of the EDI-2 were observed: −41.94 Δ% of B (baseline, 0.91 ± 1.64; 3 weeks POS, 0.53 ± 1.16), the −19.30 Δ% of DT (baseline, 9.29 ± 7.81; 3 weeks POS, 7.50 ± 7.18), the −50.45 Δ% of I (baseline, 3.26 ± 4.29; 3 weeks POS, 1.62 ± 2.85). After 3 weeks of POS treatment, in the PreOB/OB group significant differences, (p < 0.05) in terms of responses to the subscales of the EDI-2, were observed: −31.25 Δ% of B (baseline, 3.64 ± 4.12; 3 weeks POS, 2.50 ± 2.89), the −15.48 Δ% of DT (baseline, 14.09 ± 6.35; 3 weeks POS, 11.91 ± 5.76), the −36.72 Δ% of I (baseline, 5.82 ± 4.92; 3 weeks POS, 3.68 ± 4.51) (Table 2).

**Table 2:** Comparison of psychometric parameters of normal weight lean, normal weight obese and pre-obese/obese groups of women between baseline and after 3 weeks POS treatment

	NWL		NWO		PreobOB	
	Baseline	POS	Baseline	POS	Baseline	POS
EDI-2_DT	2.89 ± 4.63 (0.00–20.00)	2.94 ± 4.40 (0.00–19.00)	5.26 ± 6.33 (0.00–21.00)	5.09 ± 6.08 (0.00–20.00)	8.41 ± 7.58 (0.00–18.00)	8.50 ± 7.84 (0.00–19.00)
EDI-2_B	1.00 ± 2.45 (0.00–10.00)	0.39 ± 1.24 (0.00–5.00)	0.91 ± 1.64 (0.00–7.00)	0.53 ± 1.16^a^ (0.00–5.00)	3.64 ± 4.12 (0.00–16.00)	2.50 ± 2.89^b^ (0.00–10.00)
EDI-2_BD	5.28 ± 6.09 (0.00–23.00)	4.94 ± 6.01 (0.00–23.00)	9.29 ± 7.81 (0.00–27.00)	7.50 ± 7.18^a^ (0.00–25.00)	14.09 ± 6.35 (2.00–24.00)	11.91 ± 5.76^b^ (0.00–20.00)
EDI-2_I	1.39 ± 2.20 (0.00–6.00)	1.22 ± 1.90 (0.00–5.00)	3.26 ± 4.29 (0.00–17.00)	1.62 ± 2.85^a^ (0.00–10.00)	5.82 ± 4.92 (1.00–19.00)	3.68 ± 4.51^b^ (0.00–16.00)
EDI-2_P	3.83 ± 3.54 (0.00–14.00)	3.61 ± 3.16 (0.00–13.00)	4.85 ± 3.00 (0.00–11.00)	4.74 ± 2.99 (0.00–12.00)	3.36 ± 3.67 (0.00–14.00)	3.32 ± 3.81 (0.00–15.00)
EDI-2_ID	2.33 ± 2.47 (0.00–9.00)	2.06 ± 2.39 (0.00–9.00)	3.53 ± 3.55 (0.00–16.00)	3.47 ± 3.67 (0.00–17.00)	3.68 ± 3.37 (0.00–10.00)	3.59 ± 3.45 (0.00–10.00)
EDI-2_IA	3.61 ± 3.96 (0.00–12.00)	3.22 ± 3.61 (0.00–11.00)	4.47 ± 5.93 (0.00–23.00)	4.41 ± 6.03 (0.00–24.00)	5.36 ± 5.02 (0.00–16.00)	5.27 ± 4.73 (0.00–15.00)
EDI-2_MF	7.89 ± 5.14 (1.00–21.00)	7.67 ± 4.98 (1.00–20.00)	5.76 ± 3.24 (0.00–14.00)	5.59 ± 3.28 (0.00–15.00)	5.50 ± 4.59 (1.00–20.00)	5.45 ± 4.27 (1.00–19.00)
EDI-2_A	3.50 ± 2.46 (0.00–8.00)	3.56 ± 2.50 (0.00–9.00)	3.53 ± 1.97 (0.00–7.00)	3.38 ± 1.97 (0.00–8.00)	4.50 ± 2.09 (1.00–8.00)	4.27 ± 2.07 (0.00–7.00)
EDI-2_IR	1.61 ± 2.48 (0.00–9.00)	1.67 ± 2.28 (0.00–8.00)	3.50 ± 3.86 (0.00–14.00)	3.68 ± 4.09 (0.00–15.00)	2.32 ± 2.92 (0.00–9.00)	2.27 ± 2.81 (0.00–9.00)
EDI-2_SI	2.39 ± 2.17 (0.00–7.00)	2.33 ± 2.00 (0.00–6.00)	4.03 ± 3.38 (0.00–15.00)	3.94 ± 3.20 (0.00–14.00)	4.00 ± 3.41 (0.00–14.00)	3.91 ± 3.38 (0.00–15.00)
SCL90R_Som	0.67 ± 0.12 (0.50–0.83)	0.30 ± 0.15^c^ (0.08–0.50)	0.61 ± 0.35 (0.33–1.08)	0.28 ± 0.11^a^ (0.17–0.42)	0.87 ± 0.63 (0.08–1.67)	0.42 ± 0.26^b^ (0.17–0.67)
SCL90R_Obs Comp	0.81 ± 0.36 (0.50–1.30)	0.37 ± 0.13^c^ (0.30–0.60)	0.60 ± 0.23 (0.30–0.80)	0.27 ± 0.10^a^ (0.20–0.40)	0.46 ± 0.34 (0.20–1.00)	0.20 ± 0.00^b^ (0.20–0.20)
SCL90R_Interp Sens	0.75 ± 0.41 (0.22–1.33)	0.22 ± 0.23^c^ (0.00–0.56)	0.56 ± 0.09 (0.44–0.67)	0.26 ± 0.11^a^ (0.11–0.33)	0.45 ± 0.29 (0.11–0.89)	0.56 ± 0.00^b^ (0.56–0.56)
SCL90R_Dep	0.70 ± 0.35 (0.38–1.31)	0.27 ± 0.26^c^ (0.00–0.69)	0.18 ± 0.04 (0.15–0.23)	0.26 ± 0.10 (0.15–0.38)	0.52 ± 0.67 (0.08–1.62)	0.23 ± 0.24 (0.00–0.46)
SCL90R_Anx	0.56 ± 0.34 (0.12–1.00)	0.17 ± 0.15 (0.10–0.43)	0.30 ± 0.22 (0.04–0.57)	0.31 ± 0.14 (0.21–0.50)	0.66 ± 0.42 (0.03–1.19)	0.08 ± 0.08^b^ (0.00–0.16)
SCL90R_Anger Host	0.50 ± 0.22 (0.17–0.67)	0.20 ± 0.07^c^ (0.17–0.33)	0.39 ± 0.16 (0.17–0.50)	0.17 ± 0.14^a^ (0.00–0.33)	0.78 ± 0.49 (0.00–1.33)	0.17 ± 0.17^b^ (0.00–0.33)
SCL90R_Phob	0.10 ± 0.17 (0.00–0.43)	0.06 ± 0.13 (0.00–0.29)	0.33 ± 0.19 (0.14–0.57)	0.19 ± 0.07^a^ (0.14–0.29)	0.37 ± 0.56 (0.00–1.29)	0.07 ± 0.07^b^ (0.00–0.14)
SCL90R_Paran	0.82 ± 0.63 (0.00–1.67)	0.31 ± 0.27^c^ (0.00–0.67)	0.17 ± 0.25 (0.00–0.50)	0.00 ± 0.00^a^ (0.00–0.00)	0.39 ± 0.32 (0.00–0.83)	0.17 ± 0.17^b^ (0.00–0.33)
SCL90R_Psych	0.26 ± 0.18 (0.10–0.60)	0.06 ± 0.08 (0.00–0.18)	0.26 ± 0.17 (0.11–0.49)	0.15 ± 0.13^a^ (0.00–0.31)	0.34 ± 0.40 (0.00–0.98)	0.08 ± 0.08^b^ (0.00–0.16)
SCL90R_GSI	0.58 ± 0.23 (0.29–0.92)	0.23 ± 0.07^c^ (0.17–0.32)	0.45 ± 0.20 (0.23–0.69)	0.25 ± 0.02^a^ (0.22–0.27)	0.54 ± 0.38 (0.14–1.13)	0.24 ± 0.12^b^ (0.13–0.36)
BUT_GSI	0.55 ± 0.43 (0.09–1.21)	0.39 ± 0.43^c^ (0.12–1.15)	1.11 ± 0.66 (0.24–1.71)	0.79 ± 0.64 (0.15–1.62)	1.08 ± 0.69 (0.41–2.09)	0.54 ± 0.41^b^ (0.15–0.94)
BUT_WP	0.93 ± 0.82 (0.13–2.38)	0.79 ± 1.05 (0.13–2.63)	1.96 ± 1.17 (0.38–2.88)	1.42 ± 1.27 (0.38–3.13)	1.97 ± 0.60 (1.13–2.75)	1.13 ± 0.65^b^ (0.50–1.75)
BUT_BIC	0.58 ± 0.62 (0.11–1.56)	0.37 ± 0.56^c^ (0.00–1.33)	1.48 ± 0.85 (0.44–2.44)	1.00 ± 0.78 (0.22–2.00)	1.27 ± 1.20 (0.11–3.00)	0.67 ± 0.58^b^ (0.11–1.22)
BUT_A	0.18 ± 0.20 (0.00–0.50)	0.04 ± 0.07^c^ (0.00–0.17)	0.28 ± 0.22 (0.00–0.50)	0.11 ± 0.16^a^ (0.00–0.33)	0.13 ± 0.14 (0.00–0.33)	0.17 ± 0.17^b^ (0.00–0.33)
BUT_CSM	0.71 ± 0.48 (0.00–1.33)	0.57 ± 0.30 (0.17–1.00)	1.06 ± 0.67 (0.17–1.67)	0.89 ± 0.87 (0.00–2.00)	0.99 ± 0.73 (0.17–1.83)	0.33 ± 0.35^b^ (0.00–0.67)
BUT_D	0.13 ± 0.10 (0.00–0.20)	0.00 ± 0.00^c^ (0.00–0.00)	0.13 ± 0.20 (0.00–0.40)	0.13 ± 0.20 (0.00–0.40)	0.57 ± 0.75 (0.00–1.80)	0.10 ± 0.10^b^ (0.00–0.20)

Results are expressed in mean value ± standard deviation, and minimum and maximum for each parameter. Values of p < 0.05 are considered significant

*A* asceticism, *A* avoidance, *Anger Host* anger/hostility, *Anx* anxious, *B* bulimia, *BD* body dissatisfaction, *BIC* body image concerns, *BMI* body mass index, *BUT* body uneasiness test, *CSM* compulsive self-monitoring, *D* depersonalization, *Dep* depression, *DT* drive for thinness, *EDI-2* eating disorder inventory-2, *GSI* global severity index, *I* ineffectiveness, *IA* interoceptive awareness, *IC* body image, *ID* interpersonal distrust, *IG* intervention group, *Interp Sens* interpersonal sensitivity, *IR* impulse regulation, *MF* maturity fears, *NWL* normal weight lean, *NWO* normal weight obese, *Obs* obsessive/compulsive, *P* perfectionism, *Paran* paranoia, *Phob* phobia, *POS* probiotic oral suspension, *PreOB/OB* preobese–obese, *PSDI* positive symptom distress index, *PST* positive symptom total, *Psych* psychoticism, *SCL90R* symptom checklist 90, *SI* social insecurity, *Som* somatization, WP weight phobia

^a^NWO T0 vs T1 p < 0.05; ^b^ PreOB/OB T0 vs T1 p < 0.05; ^c^ NWL T0 vs T1

A significant improvement of the orocaecal transit time and gastrointestinal symptoms were observed (p < 0.05) after 3 weeks of POS treatment respect to placebo. Moreover, significant differences were observed for meteorism (p < 0.05) and defecation frequency (p < 0.05) (data not show).

## Discussion

The link between the “somatic” and “mental” is undeniably a subject that has fascinated through the years and until the present day many artists and philosophers. Also researchers and scientists daily make their contribution to reveal this chimera showing a complex but fascinating harmonic unit.

The gut–microbiota–brain axis includes the central nervous system, neuroendocrine and neuro-immune system, the sympathetic and parasympathetic arms of the autonomic nervous system, the enteric nervous system and, most importantly, the intestinal microbiota [38]. Some bacteria within the human gastrointestinal tract have the capacity to produce many neurotransmitters and neuromodulators, such as norepinephrine, serotonin, dopamine, acetylcholine, histamine and gamma-aminobutyric acid [7]. Due to these new evidences about the fundamental role of gut microbiota in the alteration of immune, neural and endocrine pathways, the so-called “gut–brain axis” is acquiring new significance, even if the communication routes are not yet defined [38].

From clinical experience and from the literature it is clear the importance of “personalization” of treatment, also respect to the individuals we have in care, taking into account body composition, gut microbiota, feeding behaviour, attitude towards food and the presence of emotional states that may influence them. The intake of probiotic influences the secretion of molecules on which depend anxiety and depression, and simultaneously affects neuroendocrine response to stress.

Recent data show the strong correlation between dysbiosis and a variety of conditions such as obesity, allergies, autoimmune disorders, Irritable Bowel Syndrome, Inflammatory Bowel Disease and psychiatric disorders [39, 40]. *Bifidobacterium* and *Atopobium* were significantly less abundant in obese animals compared to the non-obese rats, in conjunction with significantly higher levels of the *Clostridium cluster* XIVa and *Lactobacillus* group [41]. In the meantime, Cani et al. reported a reduction in the *Clostridium cluster* XIVa (*Clostridium coccoides*) group, along with lower *Bifidobacterium* and *Bacteroides* levels in mice fed high-fat diet. An increase of *Firmicutes* levels was observed in high-fat fed mice, while Bacteroides phylum decreased overtime in obese animals [42]. Angelakis et al. [43], highlighted elevated levels of *Bacteroidetes phylum*, a strong abundance of the *Firmicutes phylum* and elevated concentrations of *Lactobacilli* in the gut microbiota of obese and overweight adults compared to lean individuals. Moreover, the supplementation with lactic acid bacteria brought to weight modification, suppression of the neuroendocrine stress response and relieved abdominal dysfunction [44, 45].

Consistent with these considerations, it has been prepared a new formulation of POS, containing different strain of bacteria, i.e. *Streptococcus thermophiles*, *Bifidobacterium animalis* subsp. *Lactis*, *Streptococcus thermophiles*, *Lactobacillus bulgaricus, Lactococcus lactis* subsp. *Lactis, Lactobacillus acidophilus, Lactobacillus plantarum, Lactobacillus reuteri*, for a total of 120 × 10^10^ colony-forming unit CFU.

We tested the effectiveness of this new POS formulation, having two main purposes: (1) to investigate the correlation between body composition and the presence of psychological disorders and psychopathological symptoms in people affected by NWO syndrome and obesity respect to normal lean individual; (2) to check whether the intake of POS could change all the examined parameters, in order to make an early diagnosis and to block any nascent development of a psychopathological disorder, taking into account all its future consequences.

In the present study, the cut-off point of total body fat was 30% [46]; the analysis of anthropometric and body composition values showed 24% of NWO, 26% of NWL and 50% of PreOB/OB women. In particular, between the NWL versus the NWO group, were highlighted several significant differences (p < 0.05), like weight, BMI, hip circumference, TBW (%), TBFat (% and g), FM (kg and %) and FFM (%), as well as in NWL versus the PreOB/OB group, in terms of weight, BMI, waist and hip circumference, PA (°), TBW (%), ECW (%), ICW (L and %), TBFat (% and g), FM (kg and %) and FFM (%) (p < 0.05).

Moreover, significant differences (p < 0.05) between the NWO and the PreOB/OB groups in terms of weight, BMI, Reactance, PA, TBW (L and %), ECW (L and %), ICW (L and %), TBFat (% and g) were observed.

As a challenge in the development of effective strategies to prevent the increased prevalence of obesity, data reported in this study highlight the efficacy of the POS treatment on body composition in subjects with more than 30% of total body fat, given the considerable improvement of BMI, FM (kg and %), hydration status as TBW, ECW and ICW, and FFM (p < 0.05) in NWO group, as well as the improvement of weight, BMI, waist, hip, resistance, PA, TBW, ICW ECW, FM, and FFM in the PreobOB group (p < 0.05), suggesting a safe and effective intervention for general population, with substantial benefits to public health.

Many neurotransmitters and neuromodulators secreted by bacteria are able to modulate the state of the host mood: gamma-aminobutyric acid is produced by certain *Lactobacillus* and *Bifidobacterium* species; norepinephrine is released by *Escherichia, Bacillus* and S*accharomyces* spp.; 5 Hydroxy Triptamine is released by *Candida*, *Streptococcus*, *Escherichia* and *Enterococcus* spp.; dopamine is produced by *Bacillus* and acetylcholine by *Lactobacillus* [47]. Dinan et al. have reported that probiotic *Bifidobacterium infantis* 35624 has shown to have an antidepressant action in preclinical models of depression acting as a psychobiotic with a mental health benefit [48]. Gut composition is affected also by the resilience to environmental stress, impairing the cortisol awaking response and emotional reaction in healthy subjects [49]. On the other hand, it has been shown that psychological stress itself leads to dysbiosis [50, 51], turning in a vicious circle.

Our results seem to confirm the high prevalence of body image disorders in NWO and PreoOB/OB patients. According to literature data, we provided the evidence that POS therapy improves the psychological state, reducing the positivity to BUT (−24.90%) and the alteration of body image perception, as demonstrated by the significant reduction in the subscales of the EDI-2 responses both in NWO than in PreOB/OB (−41.94 Δ% of B, the −19.30 Δ% of DT, the −50.45 Δ% of I in NWO; −31.25 Δ% of B, the −15.48 Δ% of DT in PreOB/OB).

It has been demonstrated that the oral administration of the probiotic *Bifidobacterium longum* NCC3001 (Morinaga, Japan) is able to prevent the anxiety-like behavior associated with gut inflammation in animals with an intact vagus nerve [51], as did *Lactobacillus rhamnosus* and *Lactobacillus hilgardii* [52]. *Lactobacillus reuteri* prevented the physiological signs of visceral pain, with a reduction in cardio-autonomic response [53], and *Bifidobacterium lactis* decreased visceral hypersensitivity when colorectal distention occurred in the context of psychological stress.

Hence, the lactulose passes intact the stomach and small intestine, and reaches the caecum, where the bacteria (normally present in the colon flora) break it down, leading to the production of hydrogen. Part of the hydrogen that forms is absorbed by the intestinal mucous and therefore enters the bloodstream before being released at the pulmonary alveoli and expired. By evaluating the time at which hydrogen appears in the breath, we are indirectly able to determine orocaecal transit time [35]. After POS treatment, a reduction of bacterial overgrowth (p < 0.05) was observed in NWO and PreOB/OB when compared to controls. After POS therapy, a statistically significant improvement was seen in the intestinal transit time in all subjects. An increase in the number of weekly defecations and a reduction of meteorism in subjects affected by constipation were recorded.

## Conclusions

A 3-week intake of selected probiotics, by modulating body composition, bacterial contamination, psychopathological scores and eating behaviour of women affected by NWO syndrome and obesity, offers a tractable approach to problems related to obesity, psychological state and unhealthy eating. One improvement of this study may be to extend the administration period of POS, as probiotics need several weeks to proliferate.

Despite the limitations of our study, related to the study design and the low sample size, our results highlighted that this new formulation of POS may possibly have potential as a therapeutic strategy for prevention and/or treatment of certain eating behaviour disorders and anxiety-like conditions, to avoid a worsening of the psychiatric symptomatology, the establishment of a global functional impairment of the subject and to improve the quality of life of patients. Moreover, these results highlight the need for a more detailed psychiatric evaluation of subjects with an alteration of body image perception, even when this alteration does not fit into a previous pattern referred as eating disorder.

Further research is needed on a larger population and for a longer period of treatment with a controlled trial before definitive conclusions can be made.

## Abbreviations

A: asceticism
A: avoidance
Anger Host: anger/hostility
Anx: anxious
B: bulimia
BCM: body cell mass
BCMI: body cell mass index
BD: body dissatisfaction
BIC: body image concerns
BMI: body mass index
BUT: body uneasiness test
CFU: colony-forming unit
CG: control group
CSM: compulsive self-monitoring
D: depersonalization
Dep: depression
DT: drive for thinness
DXA: dual X-ray absorptiometry
ECW: extracellular water
EDI-2: eating disorder inventory-2
GI: gastrointestinal
GSI: global severity index
I: ineffectiveness
IA: interoceptive awareness
IC: body image
ICW: intracellular water
ID: interpersonal distrust
IG: intervention group
Interp Sens: interpersonal sensitivity
IR: impulse regulation
LBT: lactulose breath test
LPL: lipoprotein lipase
LPS: lipopolysaccharide
MF: maturity fears
NS: not significant
NWL: normal weight lean
NWO: normal weight obese
Obs: obsessive/compulsive
P: perfectionism
PA: phase angle
Paran: paranoia
PBF: body fat percentage
Phob: phobia
POS: probiotic oral suspension
PreOB/OB: preobese–obese
PSDI: positive symptom distress index
PST: positive symptom total
Psych: psychoticism
SCL90R: symptom checklist 90
SI: social insecurity
Som: somatization
TBBone: total body bone
TBFat: total body fat
TBLean: total body lean
TBW: total body water
WP: weight phobia
